# Novel Hepatitis E Virus in Ferrets, the Netherlands

**DOI:** 10.3201/eid1808.111659

**Published:** 2012-08

**Authors:** V. Stalin Raj, Saskia L. Smits, Suzan D. Pas, Lisette B.V. Provacia, Hanneke Moorman-Roest, Albert D.M.E. Osterhaus, Bart L. Haagmans

**Affiliations:** Erasmus Medical Center, Rotterdam, the Netherlands (V. Stalin Raj, S.L. Smits, S.D. Pas, L.B.V. Provacia, A.D.M.E. Osterhaus, B.L. Haagmans);; Viroclinics Biosciences BV, Rotterdam (S.L. Smits, A.D.M.E. Osterhaus);; and Ferret Clinic Brouwhuis, Helmond, the Netherlands (H. Moorman-Roest)

**Keywords:** hepatitis E virus, viruses, ferrets, PCR, pyrosequencing, the Netherlands

**To the Editor:** Hepatitis E virus (HEV), a member of the family *Hepeviridae* and the genus *Hepevirus*, is transmitted by the fecal–oral route and causes liver inflammation, which leads to mortality rates of ≤20% in pregnant woman (*1**,**2*). Human hepatitis E is a major disease not only in developing countries but also in industrialized countries, and identification of animal strains of HEV in pigs and deer and its zoonotic potential has raised considerable public health concerns (*1**,**3*). Recent reports suggest that other animals such as rats, mongooses, chickens, rabbits, and trout also may harbor HEVs (*1**–**5*). The genomes of these viruses are ≈6.6 kb–7.2 kb and encode 3 open reading frames (ORFs) flanked by a capped 5′ end and a poly A tail at the 3′ end (*1**,**3*). We used random PCR amplification and high-throughput sequencing technology to investigate HEV sequences in ferrets (*Mustela putorius*) from the Netherlands.

In 2010, fecal samples were collected from ferrets in the Netherlands and stored at −80°C. Samples that were negative for ferret coronavirus (*6*) were further characterized for other pathogens. Viral RNA was isolated and viral metagenomic libraries were constructed for 454 pyrosequencing as described (*7**,**8*), and 248,840 sequence reads were generated from 7 fecal samples. Using Blastn and Blastx (www.ncbi.nlm.nih.gov/BLAST), we identified 289 sequence reads in 1 sample that were related to rat HEV and that could be assembled into 6 contigs covering ≈50% of the ferret HEV (FRHEV) genome.

We then developed a set of nested PCR primers on the basis of obtained sequences to detect viral RNA (Technical Appendix Table 1). Total RNA extracted from 43 ferret fecal samples collected from 19 locations in the Netherlands was used to perform reverse transcription PCR amplification. Using this PCR, we detected viral RNA in 4 (9.3%) fecal samples tested from 4 locations (distance between each sampling location ranged from 25 km to 127 km). All amplicons were confirmed by nucleotide sequencing. We have limited information regarding the clinical disease this virus may cause because these samples were obtained from household pet ferrets that did not show overt clinical signs. In addition, 4/16 animals from a single farm were IgG positive when tested for IgG against HEV by using recombinant human HEV protein (Wantai, Beijing, China).

To characterize the complete genome of this virus, we selected 2 PCR-positive samples (FRHEV4 and FRHEV20), developed different sets of specific primers on the basis of sequence fragments obtained by 454 pyrosequencing, directly sequenced amplicons by Sanger sequencing, and used a rapid amplification of cDNA ends PCR to obtain 5′ and 3′ frame end sequences. Using overlapping fragments we assembled 2 complete FRHEV genome sequences that contained 6,854 nt, including a 13-nt 3′ poly A tail and a 12-nt 5′ end. FRHEV full-genome sequences FRHEV4 and FRHEV20 showed 98.6% sequence identity and were deposited into GenBank under accession nos. JN998606 and JN998607, respectively.

The FRHEV genome contains a complete ORF1 gene that encodes a nonstructural protein of 1,596 aa, an ORF2 gene that encodes a capsid protein of 654 aa, an ORF3 gene that encodes a phosphoprotein of 108 aa, and a 3′ noncoding region of 78 nt. Sequence analyses indicated that the FRHEV genome shared the highest identity (72.3%) with rat HEV. Sequence identity with HEV genotypes 1–4 and rabbit and avian HEVs ranged from 54.5% to 60.5% (Technical Appendix Table 2). The FRHEV genome organization was found to be slightly different from other HEVs and included a putative ORF (ORF4) of 552 nt that overlapped with ORF1 (Technical Appendix Figure). A similar pattern of genome organization was observed for both FRHEVs.

Phylogenetic analysis of the complete genomes clearly showed that FRHEV was separated from genotype 1–4 HEVs and clustered with rat HEV (Figure). Similar phylogenetic clustering was observed when nucleotide and deduced amino acid sequences of ORF1, ORF2, and ORF3 were analyzed separately. The phylogenetic distance between rat HEV and FRHEV is larger than the distance between genotype 1 and genotype 2 HEV.

**Figure:**
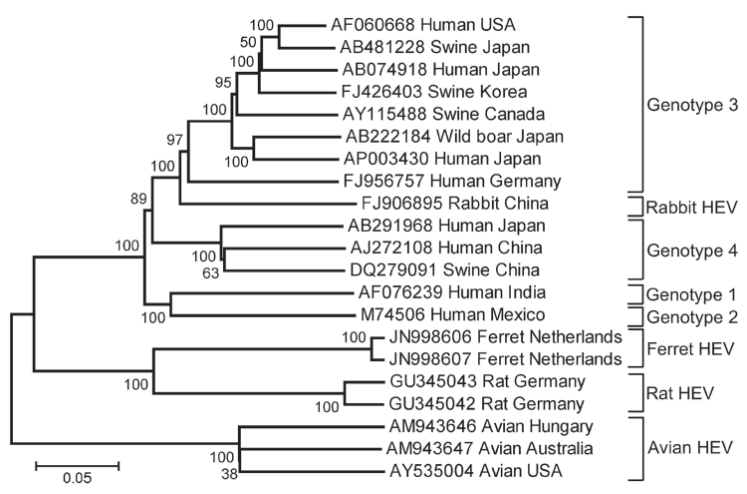
Phylogenetic tree based on the complete genomic sequences of ferret hepatitis E viruses (HEVs) and human, rabbit, swine, avian, and rat HEV strains. Names of HEV strains follow GenBank accession numbers. Sequence alignment was performed by using ClustalW in the MEGA5.0 software package (www.megasoftware.net), and the trees were constructed by using the neighbor-joining method with p-distance (gap/missing data treatment; complete deletion) and 1,000 bootstrap replicates as in MEGA version 5.0. Scale bar indicates nucleotide substitutions per site.

In recent years, an increasing number of sporadic cases of hepatitis E have been reported (*1**,**9*). Several observations suggest that autochthonous cases are caused by zoonotic spread of infection from wild or domestic animals (*1**,**3**,**9*). In addition, IgG anti-HEV seropositivity in the United States has been associated with several factors, including having a pet at home (*10*). Further studies are needed to identify the zoonotic potential of FRHEV.

Technical Appendix Genome organization of hepatitis E viruses (HEVs) and initiation of translation of open reading frame 1 (ORF1), ORF2, and ORF3 of ferret HEV.
