# Supplementary material for: Novel Hepatitis E Virus in Ferrets, the Netherlands
Source: Emerg Infect Dis. 2012 Aug;18(8):1369–70. doi: 10.3201/eid1808.111659 (PMC3414025; doi:10.3201/eid1808.111659)
Supplement: Technical Appendix — Genome organization of hepatitis E viruses (HEVs) and initiation of translation of open reading frame 1 (ORF1), ORF2, and ORF3 of ferret HEV. [file 11-1659_Techapp-s1.pdf]

Article DOI: http://dx.doi.org/10.3201/eid1808.111659

Novel Hepatitis E Virus in Ferrets, the Netherlands

Technical Appendix

Table 1. Primers used for amplification and sequencing of the ferret HEV genome*

Primer	Sequence, 5′ → 3′	Nucleotide position†
FRHEV-F1	GGCTGGCGTTTGCTTGGAGG	256–275
FRHEV-R1	TTCGAATCCAACGCTGGTGAC	1158–1178
FRHEV-F2	GTACTATCACGGCCAATGAG	1077–1096
FRHEV-R2	CAGCCTATAGGGCATAGTAAG	1681–1701
FRHEV-F3	GCCCTGACCTTGGAGCTGAC	1592–1611
FRHEV-R3	CTATTGGCGGCGTTAACTAG	2078–2097
FRHEV-F4	GAGCTTTTGCCGGATGGGTC	2015–2034
FRHEV-R4	CACTAGCCCCTTATGGTCGA	2922–2941
FRHEV-F5	CTGTTGCCAAATCGGTCGTA	2865–2884
FRHEV-R5	GCATCAGACACGCCCAGCTC	3308–3327
FRHEV-F6	TGCCTTGACGCGCCACACTG	3250–369
FRHEV-PCR-InR	CTCACCKGAATGCTTCTTCC	4272–4291
FRHEV-PCR-ExF	TCCAGAAGGACTGCAACAAG	3876–3895
FRHEV-R7	CTCACCACGTCAGGAACAACC	4525–4545
FRHEV-F8	AGTTGAAGGTGGCGTTCCAC	4461–4480
FRHEV-R8	GCGCCTCGAGAATCGTAATC	5183–5202
FRHEV-F9	CGTGTTCCCGATGCCGGGCA	5114–5133
FRHEV-R9	CAGGCGAAGGCGTCGGCC	6164–6181
FRHEV3′RACE-ExF	TATACCTCTGTTGAGGCTGC	6049–6068
FRHEV3′RACE-InF	CCTTGGACTTTCGTCTGTTAC	6108–6128
FRHEV-5′ end-F‡	GACCATCTAGCGACCTCCACTTTTTTTTTTTTTTTTTTTT	NA
FRHEV-5′ end-R	TGCATATCTCGACCGTCAGG	338–357
FRHEV-R10	TATTTGCTGGACCCCTTC	380–397
FRHEV-PCR-ExR	CAAGATCACCGCCATGTTCCA	4319–4339
FRHEV-PCR-InF	GTCAGGGCATWTCRGCSTGG	3933–3952

*HEV, hepatis E virus; FRHEV, ferret HEV; F, forward; R, reverse; RACE, rapid amplification of cDNA ends; NA, not applicable.
†Positions of primers located in the complete genome are shown according to ferret HEV strains.
‡Nonviral primer.

Table 2. Nucleotide and amino acid sequence identities between rat, human, rabbit, and avian HEV strains compared with ferret HEV*

Strain, GenBank accession no.	% Identity with ferret HEV							
	FRHEV 4, NJ998606				FRHEV 20, NJ998607			
	Genome, nt	ORF, aa	ORF2, aa	ORF3, aa	Genome, nt	ORF1, aa	ORF 2, aa	ORF 3, aa
RatHEV, GU345042	72.2	77.1	81.4	44.4	72.3	77.2	82.3	45.7
Genotype 1,† AF076239	59.2	53.9	60.1	19.8	59.4	54.0	60.6	21.0
Genotype 2,† M745056	58.7	54.0	60.6	19.8	58.8	61.7	61.0	21.0
Genotype 3,† AF060668	60.6	55.7	61.0	18.5	60.5	55.8	61.7	19.8
Genotype 4,† AJ272108	58.8	54.7	60.5	17.3	58.9	54.9	61.2	17.3
Rabbit HEV, FJ906895	59.8	54.9	60.1	17.3	59.8	54.9	60.8	18.5
Avian HEV, AM943646	54.5	46.9	46.0	21.0	54.5	46.9	46.5	19.8

*HEV, hepatitis E virus; FRHEV, ferret HEV; ORF, open reading frame.
†Human strains.

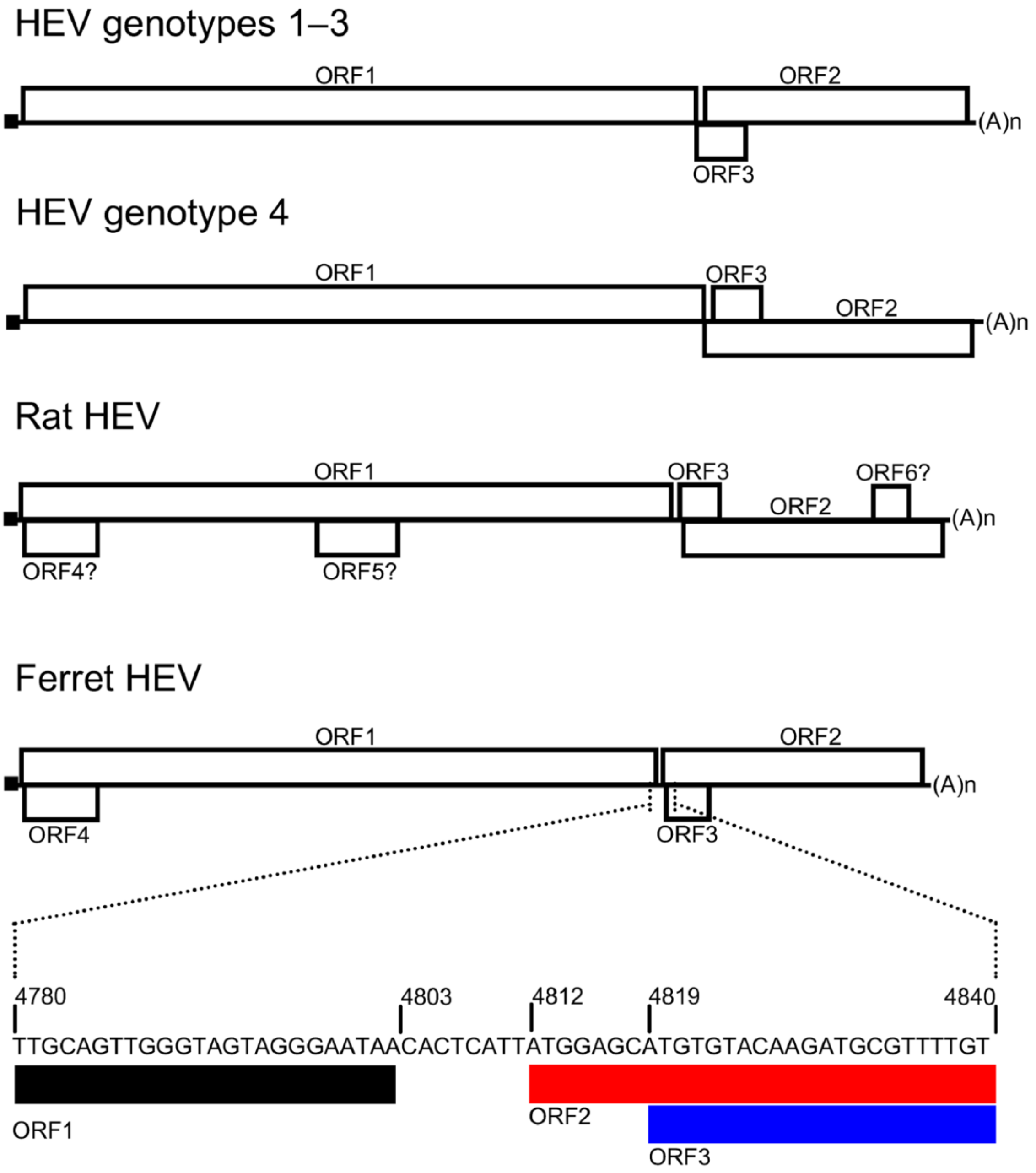

Technical Appendix Figure. Genome organization of hepatitis E viruses (HEVs) and initiation of translation of open reading frame 1 (ORF1) (black bar), ORF2 (red bar), and ORF3 (blue bar) of ferret HEV.
